# Editorial: Nanotechnological Advances in Biosensors

**DOI:** 10.3390/s91108907

**Published:** 2009-11-09

**Authors:** Jay Nadeau

**Affiliations:** Biomedical Engineering, Lyman Duff Medical Building, Room 310, 3775 University St., Montreal, QC H3A 2B4, Canada; E-Mail: jay.nadeau@mcgill.ca

A biosensor is a physicochemical or hybrid physical-chemical-biological device that detects a biological molecule, organism, or process. Because of the nature of their targets, biosensors need to be faster, smaller, more sensitive, and more specific than nearly all of their physicochemical counterparts or the traditional methods that they are designed to replace. Speed is of the essence in medical diagnosis as it permits for rapid, accurate treatment and does not allow patients to be lost to follow-up. Small size and greater sensitivity mean less-invasive sampling and detection of molecules such as neurotransmitters or hormones at biologically-relevant levels. Greater specificity allows assays to be performed in complex fluids such as blood or urine without false negative or false positive results.

Nanotechnology promises to improve biosensing on all of these fronts. Nanofabricated materials can bind directly to biomolecules and/or act as transducers to extremely small and sensitive detectors. Their sensing mechanisms can be sensitive at the single-molecule level, and include standard outputs such as fluorescence and color as well as label-free techniques such as evanescent wave coupling or electrochemistry.

This Special Issue reviews and introduces some ways in which nanofabrication and nanomaterials can aid in specific biomolecule detection. Several of the papers present complete lab-on-chip systems for microfluidic sample delivery and analysis. Germano *et al.* [1] present a biochip that works on the principle of magnetoresistive sensing. Magnetically-tagged targets can be detected down to fM concentrations. A full prototype of the sensor platform is described, including sensing and processing modules (incorporating electric and magnetic drive, signal processing, and digitalization), communication modules, and an analyzer module coupled to a computer. Assadollahi *et al.* [2] improve the speed and sensitivity of lateral flow devices by creating a microfluidic “dipstick” tester with a readout panel consisting of functionalized Au or Pd nanoparticles. Resonance-enhanced absorption (REA) of these metal particles was used to detect specific binding and could be further amplified with silver stain for increased sensitivity. The device was designed to handle blood or urine. Huang *et al.* [3] have developed a microfluidic device that amplifies the surface plasmon signal from Au nanoparticles using grooved optical fibers. Binding of an analyte to the functionalized Au particles causes a disruption of the evanescent field and thus a signal, even for analytes that are transparent at the wavelengths measured (usually UV-Visible absorption).

Principles of microfabrication for improved sensors are discussed by Passaro *et al.* [4], who model the parameters needed to use slot waveguides as sensors for environmental chemicals. Viegas *et al.* [5] present a theoretical consideration of long period fiber gratings as transducers, and demonstrate their utility by functionalizing with porous SiO_2_ nanospheres as a humidity sensor. Prakash *et al.* [6] review the different substrates that can be used to immobilize a particular enzyme (catalase), thus demonstrating the variety of issues involved in transducing an electron-transfer signal from a protein.

Other papers evaluate the potential of novel materials, particularly nanoparticles, to serve as biosensors. Three papers in this issue discuss biosensing using fluorescent semiconductor nanoparticles (quantum dots). Orcutt *et al.* [7] contribute an original article demonstrating how the stability of quantum dot fluorescence can be used to label cyanobacteria, whose autofluroescence (in both the blue and the red) has always made traditional techniques difficult. With quantum dot labeling, the intrinsic pigments can be photobleached before the signal from the quantum dots has faded. Frasco *et al.* [8] provide a unique and thorough review of how the modulation of quantum dot fluorescence by quenching and resonance energy transfer (FRET, BRET, PET) can be used to create sensors for pH, specific ions, pesticides, DNA, and specific enzymatic processes. They show the different possible conjugation and immobilization strategies for sensing in solution and on surfaces, including a detailed comparison of the many possible schemes for nucleic acid detection. Martin-Palma *et al.* [9] have written a second comprehensive review of quantum dot-based biosensors. They discuss the photophysical properties of semiconductor quantum dots made of various materials and discuss their bioconjugation. They summarize the literature on cell uptake and toxicity and discuss emerging sensing mechanisms such as cleavage of quenchers and electrochemical displacement assays. They also discuss how the multiple emission wavelengths of quantum dots can be used in multiplexed assays.

Not all nanoparticles show photoluminescence, but their optical properties may still be useful for biosensing. Kim *et al.* [10] show that localized surface plasmon resonance (LPSR) can be used to detect biological binding on gold nano-islands. In order to increase the sensitivity of the technique, they functionalize their ligands with gold nanoparticles, allowing their target receptors to be large proteins (such as streptavidin). Koh *et al.* [11] review the physics of magnetic nanoparticles and their use as relaxation switch assay sensors, relaxation sensors, and magnetoresistive sensors. They illustrate the possible approaches to sensing of biological targets and summarize the latest results using the different sensing mechanisms on a variety of agents (viruses, bacteria, cancer cells). Qi *et al.* [12] review electrogenerated chemiluminescence (ECL) in biosensors. They review the principle of ECL detection and discuss the types of nanoparticles for which it is relevant and the procedures to biofunctionalize them and immbolize them on electrodes. These principles are illustrated by Piao *et al.* [13] who demonstrate an ECL biosensor for ethanol made of carboxylate-functionalized single-wall carbon nanotubes and Au nanoparticles.

Liu *et al.* [14] review how many of these types of sensors can be applied to a single biological problem: DNA hybridization. Because DNA is self-complementary and its strands can be denatured under relatively mild conditions, and because specific oligonucleotides are easy to manufacture, probing for specific DNA sequences is one of the most successful forms of biosensing. It is also extraordinarily useful, allowing for identification of pathogens in clinical samples; of organisms in environmental communities; and of alterations of gene sequence and/or expression levels in health and disease. The authors show how the signal from fluorescently-labeled probes can be enhanced by semiconductor nanoparticles, nanoscaled metal oxide films, and CdS “nano-walnuts.” They then review the literature on methods of hybridization detection using quantum dots, Au nanoparticles, and carbon nanotubes. The detection methods and sensitivities of each method, and the improvement over non-nanoscaled materials, are summarized in a table that is sure to be of use for anyone seeking to improve sensors or to choose a sensor for a DNA-based application.

Finally, Rezek *et al.* [15] attack a challenging problem in biointerfacing by patterning human osteoblastic cells into microarrays. Such patterned growth has relevance for tissue engineering as well as complex forms of biosensing using entire living cells as transducers.

This collection gives a flavor of the many challenges faced in biosensing: functionalization, signal transduction, nonspecific interactions, and practical system integration. Many of the techniques and approaches have broad application and will be able to be adopted by multiple laboratories. Others represent complex systems that will be packaged into commercial devices that replace benchtop instruments with smaller, lighter, possibly field-ready sensors. Our hope is that the successes as well as the limitations of these sensors, and the general principles upon which they are based, can inspire further innovation in this rapidly-expanding field.
